# Supplementary material for: Identification and Characterization of the MIKC-Type MADS-Box Gene Family in Brassica napus and Its Role in Floral Transition
Source: Int J Mol Sci. 2022 Apr 13;23(8):4289. doi: 10.3390/ijms23084289 (PMC9026197; doi:10.3390/ijms23084289)
Supplement: Supplementary file 1 [file ijms-23-04289-s001.zip › ijms-1640608-supplementary/Supplementary File/IJMS Supplementary data.pdf]

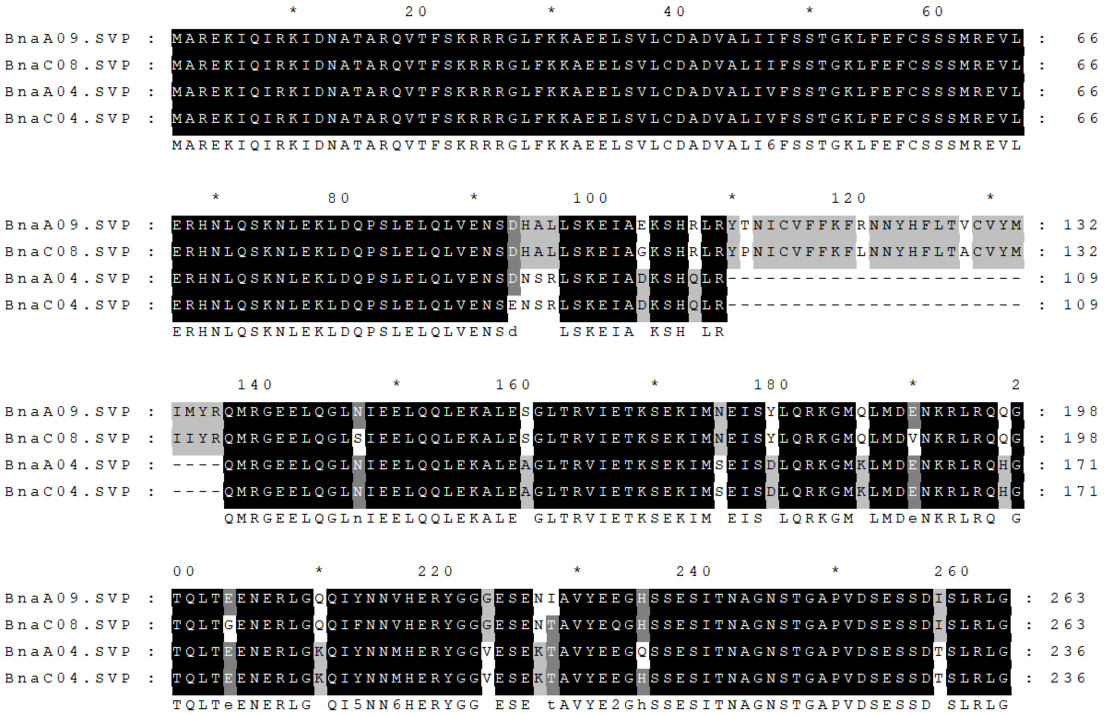

Figure S1. Amino acid sequence similarity analysis of four copies of *BnaSVP*.

(a)

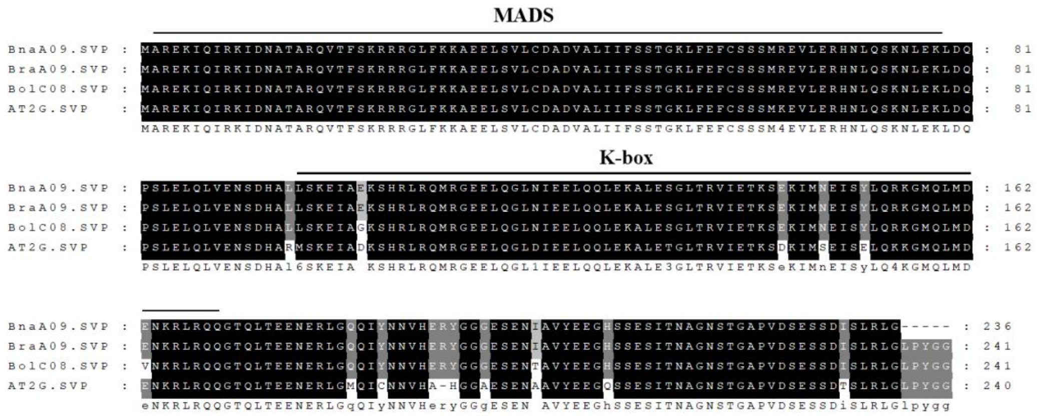

(b)

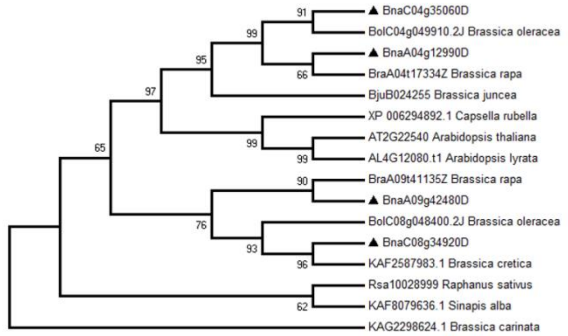

Figure S2. Domain and phylogenetic tree analysis of the *BnaSVP* gene. (a) Alignment of *SVP* homolog sequences identified from *Brassica napus* (*BnaA09.SVP*), *Brassica rapa* (*BraA09.SVP*), *Brassica oleracea* (*BolC09.SVP*), and *Arabidopsis thaliana* (*AT2G22540*). The underlined fragment represents the MADS and K-box domain; the base differences are highlighted in white. (b) Phylogenetic tree showing the sequence relationship among *SVP* homologs identified from different plants; triangle represents the homologous copy of *SVP*.

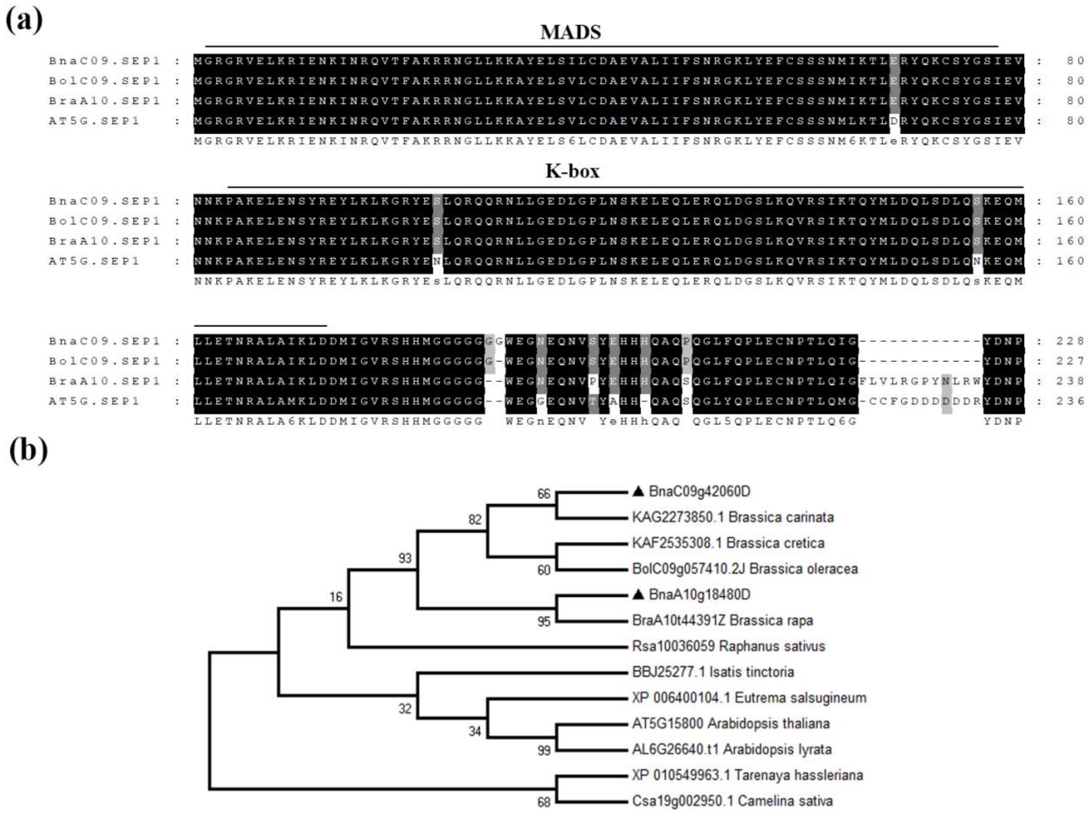

Figure S3. Domain and phylogenetic tree analysis of the *BnaSEP1* gene. (a) Alignment of the *SEP1* homolog sequences identified from *Brassica napus* (*BnaC09.SEP1*), *Brassica rapa* (*BraA10.SEP1*), *Brassica oleracea* (*BolC09.SEP1*), and *Arabidopsis thaliana* (*AT5G15800*). The underlined fragment represents the MADS and K-box domain; the base differences are highlighted in white. (b) Phylogenetic tree showing the sequence relationship among *SEP1* homologs identified from differentplants; triangle represents the homologous copy of *SEP1*.

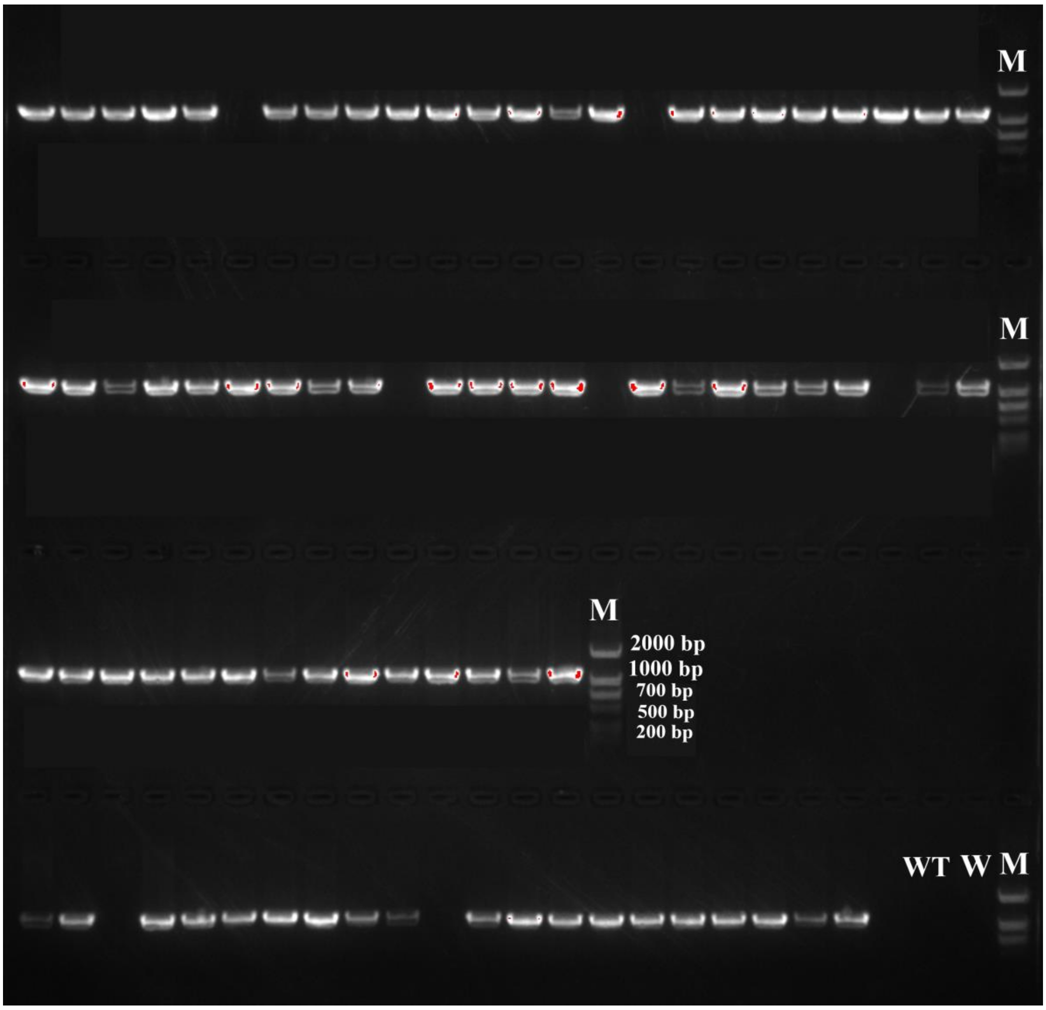

Figure S4. The positive detection in T0 regenerated plants via PCR using the Cas9-F/Cas9-R primers. Wild type (WT) and water (W) as controls; the marker (M) was provided by the laboratory. The first three lines represent the *SVP* test samples, and the fourth line represents the *SEP1* test sample.

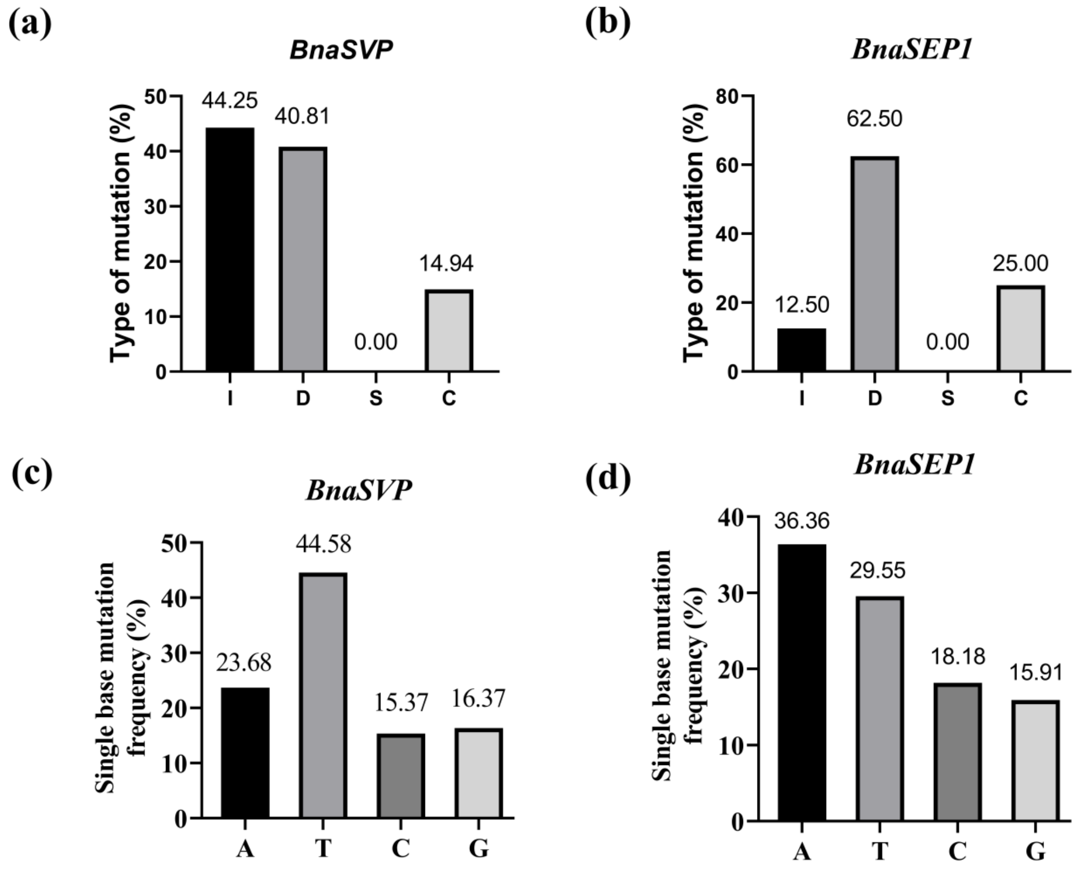

Figure S5. Mutation characteristics of *BnaSVP* and *BnaSEP1*. (a-b) The mutation types of *BnaSVP* and *BnaSEP1* are divided into insertion, deletion, base substitution, and chimeric mutations. Two or more of the above four mutations are called chimeric mutations. (c-d) Frequency of the ATCG base mutation in *BnaSVP* and *BnaSEP*.

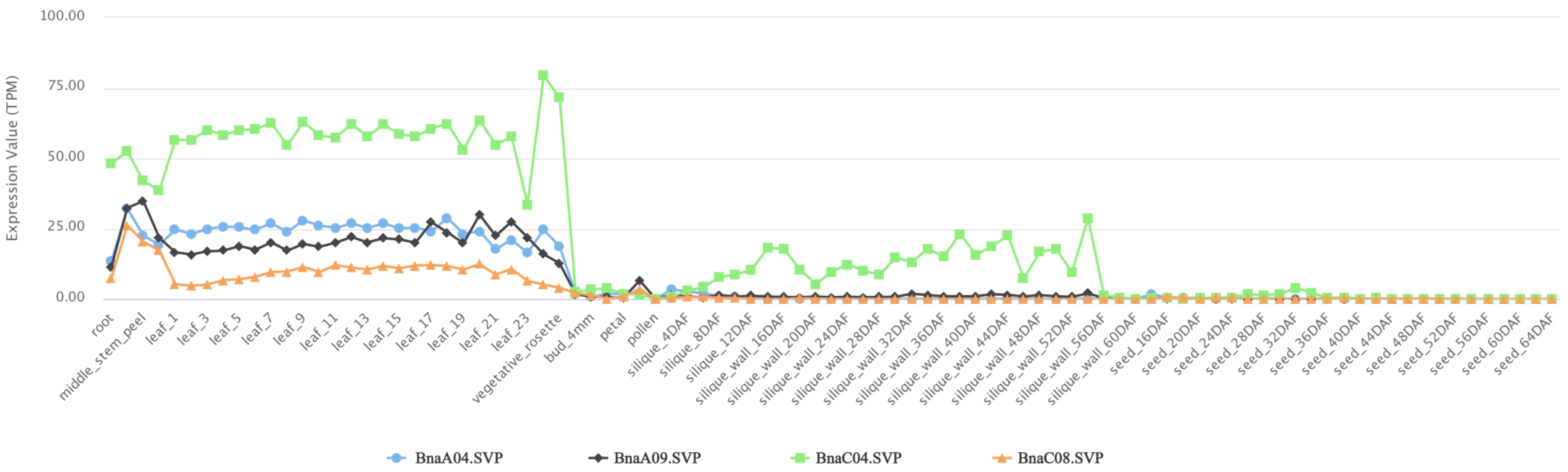

Figure S6. Expression pattern of *BnaSVP* in rapeseed. Gene expression of *BnaSVP* in various tissues. The transcriptome data were derived from the public online transcriptome platform (http://yanglab.hzau.edu.cn/BnTIR).

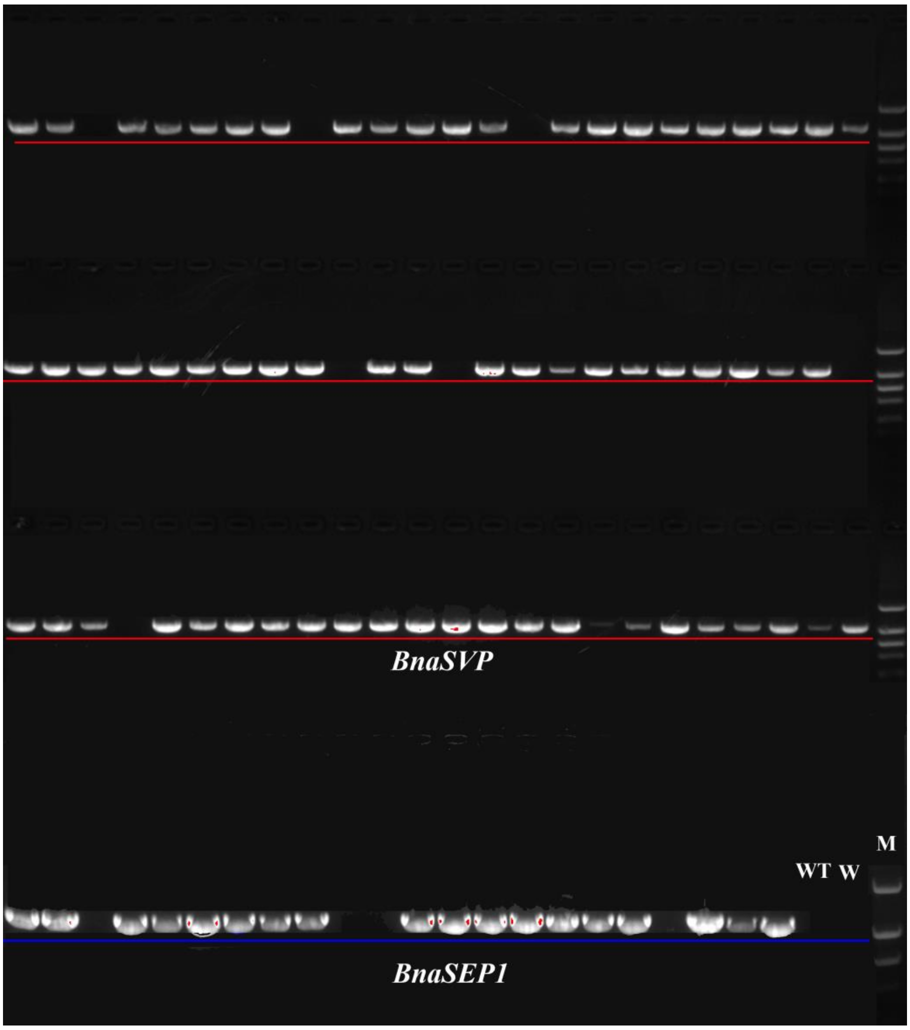

Figure S7. T-DNA free detection in T1 regenerated plants via PCR using the Cas9-F/Cas9-R primers. Wild type (WT) and water (W) as controls; the marker (M) was provided by the laboratory. The first three lines represent the *SVP* test samples, and the fourth line represents. the *SEP1* test sample.
